# Spontaneous Generation

**Published:** 1891-04

**Authors:** 


### Spontaneous Generation.

In an article upon this subject in the first number of the *Bacteriological World* the editor reaches the following conclusions in regard to micro-organisms:

1. That the words germs, bacteria, microbe, schizomycetes are used in our present literature almost as synonymous terms, but microbe seems preferable to germ or bacteria, and schizomycetes is a better scientific term than either.

2. That these are unicellular, and assimilate nourishment, seemingly by absorption in the media in which they live.

3. That they must transform, alter the foods found proper, and yet unfit in nature, for their use and appropriation.

4. That bacteria living on dead matter encounters no living resistance, while those feeding on living tissues, meet the living cells of the body and have to combat them.

5. That the diastases secreted by the various beings, whether highly organized or unicullular and microscopic, have something in common as to their respective objects, and their properties of transforming matter.

6. That the role of microbes in the world is complex and necessary, though some are injurious. They act as scavengers, return to the air and water the organizable elements abstracted daily by the vegetables of the globe, and indirectly by animals, and indispensable to life.

7. That the bacteria that invade living organisms which happen to be fit for their nourishment and growth, are in a sense parasites just as much as the tapeworm is.

8. That spontaneous generation of living organisms, no matter how little, is a fallacy.

